# Co-existence of OXA-48 and NDM-1 in colistin resistant *Pseudomonas aeruginosa* ST235

**DOI:** 10.1080/22221751.2020.1713025

**Published:** 2020-01-21

**Authors:** Cansel Vatansever, Sirin Menekse, Ozlem Dogan, Lal Sude Gucer, Berna Ozer, Onder Ergonul, Fusun Can

**Affiliations:** a Department of Infectious Diseases and Clinical Microbiology, Koc University School of Medicine, Istanbul, Turkey; b Department of Infectious Diseases and Clinical Microbiology, Kosuyolu State Hospital, Istanbul, Turkey

**Keywords:** *P. aeruginosa*, colistin resistance, high-risk clone, ST235, OXA-48, NDM-1

## Abstract

Here, we presented 11 cases with colistin-resistant *Pseudomonas aeruginosa* infection and co-existence of OXA-48 and NDM-1 in the ST235 high-risk clone. The molecular analyses were performed by Sanger sequencing and RT–PCR. The eight patients (72.7%) had an invasive infection and three (27.3%) had colonization. The 30-day mortality rate was 87.5% (7/8). Three patients (37.5%, 3/8) received colistin therapy before isolation of *P. aeruginosa*. In the Multilocus sequence typing (MLST) analysis of 11 isolates, eight (72.7%) isolates belonged to *P. aeruginosa* ST235 clone. All isolates were NDM-1 positive, and nine isolates (81.8%) were found to be positive for both OXA-48 and NDM-1. Sequences of *pmrAB* and *phoPQ* revealed numerous insertions and deletions in all isolates. In 10 isolates *pmrAB* and *phoPQ* were found to be upregulated. In conclusion, the co-existence of OXA-48 and NDM-1 genes in colistin-resistant *P. aeruginosa* ST235 high-risk clone indicates the spread of carbapenemases in clinical isolates and highlights need of continuous surveillance for high-risk clones of *P. aeruginosa*.

*Pseudomonas aeruginosa (P. aeruginosa)* is one of the most common causes of healthcare-related infections [1]. The ST235 high-risk clone of *P. aeruginosa* has high capacity to acquire antibiotic resistance and is disseminating worldwide. The ST235 clone harbours nearly 39 types of beta-lactamases especially IMP, NDM and VIM type Metallo-β-lactamases (MBLs) [2]. However, up to date, there is no report on co-existence of NDM-1 and OXA-48 in *P. aeruginosa*. Colistin resistance among *P. aeruginosa* is rare (<1%) in Europe [3]. However, it was found to be 7.4% in Korea, and 8.8% in Turkey [4,5]. Dissemination of colistin resistance in high-risk clones is concerning because of increased fatality and lack of antimicrobial therapy options. The overexpression of *phoPQ* and *pmrAB* two-component regulatory systems contribute colistin resistance by reducing the negative charge of the outer membrane in *P. aeruginosa* [6].

In this correspondence, we presented 11 cases with colistin-resistant *P. aeruginosa* infection and reported the presence of OXA-48 along with NDM-1 in the isolates belonging to the ST235 high-risk clone. We also analysed mutations and expressions of *phoPQ* and *pmrAB* systems in 11 colistin-resistant *P. aeruginosa* isolates.

Patients who were diagnosed with colistin-resistant *P. aeruginosa* infection or colonization in the ICU unit of a Cardio-Pulmonary Surgery Hospital in Istanbul between July 2017 and December 2018 were included in the study. The demographic and clinical data were recorded on a standardized case form. The patients were followed up for clinical outcomes.

The colistin resistance was determined by the broth microdilution method according to EUCAST criteria [7]. In strain typing, Multilocus sequence typing (MLST) was performed by amplifying seven housekeeping genes, according to the protocol on Pseudomonas aeruginosa MLST website (https://pubmlst.org/P.aeruginosa/). Allelic profiles and sequence types (STs) were determined using Applied Math Bionumerics V7.6 software. Clonal relatedness was determined by the repetitive PCR (rep-PCR) (Diversilab, Biomerieux). Isolates that had a similarity index >95% were considered as clonally related. Carbapenem and colistin resistance genes *(bla*_IMP_, *bla*_VIM_, *bla*_OXA_, *bla*_NDM_, *bla*_KPC_, *mcr-1*) were screened by PCR using primers as described previously, and the amplicons were confirmed by sequencing (6). For colistin resistance mechanisms, mutations in *pmrAB* and *phoPQ* were detected by Sanger Sequencing [6]. Expressions of *pmrA* and *phoP* were studied by qRT-PCR [6]. The *rpsl* was selected for normalization and *P. aeruginosa* ATCC 27853 was for calibration.

Among 11 patients, eight (72.7%) had an invasive infection and three (27.3%) had colonization with colistin-resistant *P. aeruginosa*. Eight (72.7%) patients stayed in ICU and six (54.5%) had lung transplantation. Sepsis was diagnosed in three (37.5%) of eight patients. The 30-day mortality rate was 88% (7/8). The overall mortality of *P. aeruginosa* infections in the hospital was 46% and it was 68% for carbapenem-resistant colistin susceptible *P. aeruginosa* infections. In a recent report from Spain, the overall 30-day mortality of bacteremia cases caused by ST235 was found to be 82%, however, it was 42.2% in other clones [10]. In our study, the eight of 11 (72.7%) isolates belonged to *P. aeruginosa* ST235 high-risk clone. The three isolates were identified as a novel allele (ST3078) referred by Pasteur Institutes MLST database (https://pubmlst.org/paeruginosa/)(Table 1). In Germany, the mortality rate of *P. aeruginosa* bacteremia was reported as 26% in 937 ICU units [8]. The increased mortality rates are associated with multidrug resistance [9]. The ST235 is associated with MDR or PDR profile and fatal infections [2]. In genotyping K741-K748 and K752-K753 were found to be clonally related (>95%). Other isolates belong to different clone.

**Table 1.** Clinical and laboratory characteristics of study population.

Code	ST	Source	Colistin MIC	Meropenem MIC	Carbapenemase	Empirical Therapy	Pre-exposure time to colistin	Active Therapy	Duration of Active Colistin Therapy	Survival	30-day Mortality	Clonal Relatedness
K704	235	Catheter	>64	16	OXA-48, NDM-1	Meropenem Vancomycin	0	0	0	Ex	1	
K741	235	BAL	16	16	OXA-48, NDM-1	Moxifloxacin	0	Meropenem Colistin Ertapenem	1	Ex	2	*
K740	3078	BAL	>64	8	OXA-48, NDM-1	Piperacillin Tazobactam	0	Meropenem Colistin Ertapenem	27	Ex	30	
K748	235	DTA	64	16	OXA-48, NDM-1	0	0	0	0	Discharge	Discharge	*
K752	3078	DTA	4	16	OXA-48, NDM-1	0	0	0	0	Discharge	Discharge	**
K753	3078	BAL	4	8	OXA-48, NDM-1	Piperacillin Tazobactam	0	Meropenem Colistin	14	Discharge	Discharge	**
K783	235	BAL	16	16	OXA-48, NDM-1	Meropenem Colistin	10	Meropenem Colistin	10	Ex	7	
K970	235	Catheter	32	16	NDM-1	Levofloxacin Colistin	21 days	0	0	Ex	10	
K982	235	BAL	16	16	OXA-48, NDM-1	Colistin (inhalation)	25 days	0	0	Ex	1	
K989	235	Catheter	>64	16	NDM-1	Meropenem	0	Meropenem Colistin	38	Ex	42	
K1009	235	DTA	16	16	OXA-48, NDM-1	Colistin (inhalation)	8 days	0	0	Ex	19	

Notes: BAL: Bronchoalveolar lavage; DTA: Deep tracheal aspirate.

*clone 1, **clone 2.

All isolates were found to be carbapenem-resistant. All of them were NDM-1 positive, and nine (81.8%) harbour both OXA-48 and NDM-1 beta-lactamases. Carbapenem-resistant OXA-48 positive *P. aeruginosa* was isolated in Sudan and India [11] and NDM-1 positive *P. aeruginosa* was detected in Serbia [12]. However, this is the first report of co-existence of OXA-48 and NDM-1 producing *P. aeruginosa* isolation in Turkey and Europe.

Colistin use is one of the major factors responsible for the development of colistin resistance. In our study, only four patients (36,3%) received colistin therapy before the isolation of *P. aeruginosa* (Table 1). The MICs for colistin were between 4 and >64 mg/L. In all isolates, sequences of *pmrAB* and *phoPQ* revealed numerous insertions and deletions. In the ten of them, *pmrAB* and *phoPQ* were found to be upregulated. Relative expressions of *pmrA* and *phoQ* genes were between 0.3–59.9-fold (mean 12.9-fold) and 0.9-6.9-fold (mean 4.15-fold), respectively. In nine isolates, colistin MICs and *pmrAB*-*phoPQ* expressions were found to be related (Figure 1). These results suggested that there could be additional mechanisms contributing to colistin resistance in *P. aeruginosa*.

**Figure 1.**
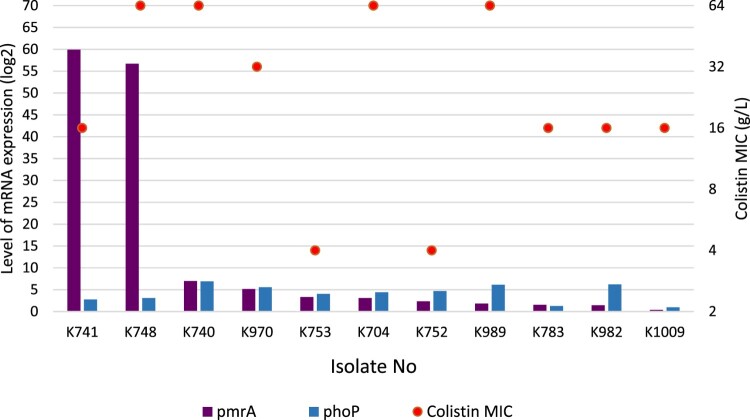
*PmrA* and *PhoP* expressions of the colistin-resistant *P. aeruginosa* in correlation with colistin MIC values.

In conclusion, colistin resistance is emerging in *P. aeruginosa* ST235 global high-risk clone. The co-existence of OXA-48 and NDM-1 genes in colistin-resistant *P. aeruginosa* ST235 high-risk clone indicates the spread of carbapenemases in clinical isolates and highlights need of continuous surveillance for high-risk clones of *P. aeruginosa*.
